# DER-YOLO: A Lightweight Stage-Wise Feature Calibration Network for Onboard Real-Time Small-Object Detection

**DOI:** 10.3390/s26144462

**Published:** 2026-07-14

**Authors:** Jiapei Wei, Azizan As’arry, Khairil Anas Md Rezali, Mohd Zuhri Mohamed Yusoff, Masnida Hussin, Tong Mu

**Affiliations:** 1 Department of Automotive Engineering, School of Mechanical and Electrical Engineering, Tianshui Normal University, Tianshui 741000, China; 2 Department of Mechanical and Manufacturing Engineering, Faculty of Engineering, Universiti Putra Malaysia, Serdang 43400, Malaysia; 3 Laboratory of Biocomposite Technology, Institute of Tropical Forestry and Forest Products, Universiti Putra Malaysia, Serdang 43400, Malaysia; 4 Department of Communication Technology and Network, Faculty of Computer Science and Information Technology, Universiti Putra Malaysia, Serdang 43400, Malaysia

**Keywords:** object detection, YOLO11, small-object detection, onboard traffic perception, autonomous driving, real-time inference

## Abstract

**Highlights:**

**What are the main findings?**

- DER-YOLO improves small-object detection in complex traffic scenes by introducing stage-wise feature calibration across the backbone, neck, and pre-head stages.
- The proposed DGC-C3k2, ECAF, and RLSK modules provide complementary gains in global context modeling, cross-scale feature fusion, and high-resolution spatial response refinement.

**What are the implications of the main findings?**

- Stage-wise feature calibration is an effective strategy for improving lightweight YOLO-based detectors without sacrificing real-time inference performance.
- DER-YOLO offers a practical lightweight solution for small-object detection in onboard vision-based traffic perception.

**Abstract:**

Accurate real-time detection of small traffic objects remains a critical challenge for onboard vision-based traffic perception, particularly under conditions of weak texture, scale variation, occlusion, and limited computational resources. To address these challenges, this paper proposes DER-YOLO, a lightweight small-object-oriented detector built upon YOLO11n, specifically designed for complex traffic scenes. DER-YOLO introduces stage-wise feature calibration across the backbone, neck, and pre-head stages to enhance small-object representation. First, a Decoupled Global Context C3k2 (DGC-C3k2) module strengthens contextual representation for weak and low-saliency traffic objects after local feature extraction. Second, an ECA-guided Cross-scale Adaptive Fusion (ECAF) module adaptively balances high-level semantic cues and shallow high-resolution details to improve multi-scale feature interaction. Third, a Refined Large Selective Kernel (RLSK) module refines high-resolution spatial responses before the P3 detection head, enhancing small-object localization. Extensive experiments on KITTI and BDD100K demonstrate that DER-YOLO improves detection accuracy while maintaining real-time inference. On KITTI, it achieves 86.95% mAP@0.5 and 60.89% mAP@0.5:0.95, with small-object AP@0.5:0.95 increasing from 27.0% to 29.3%. On BDD100K, it achieves 55.05% mAP@0.5 and 29.21% mAP@0.5:0.95, with small-object AP@0.5:0.95 increasing from 12.6% to 15.2%. With 2.744 M parameters, 7.401 GFLOPs, and over 100 FPS, DER-YOLO provides an effective and lightweight solution for real-time small-object detection in onboard traffic perception scenarios.

## 1. Introduction

Object detection is a core component of autonomous driving perception systems and provides essential visual information for downstream tasks such as path planning, obstacle avoidance, and traffic-rule decision making [1,2]. In real traffic scenes, onboard vision-based traffic perception systems must detect multiple categories of road users and traffic elements, including vehicles, pedestrians, cyclists, traffic signs, and traffic lights. These tasks are often performed under complex imaging conditions, such as illumination variation, partial occlusion, large-scale differences, background clutter, rain, fog, glare, backlighting, and nighttime low illumination [3,4]. At the same time, autonomous driving platforms impose strict requirements on inference speed, model size, and computational cost. Therefore, traffic-scene detectors must achieve a balance among detection accuracy, real-time performance, and environmental adaptability [5].

The YOLO family has become one of the most representative frameworks for real-time object detection. Since its initial release, it has undergone continuous architectural improvements, progressing from early versions such as YOLOv1 [6] to more recent variants including YOLO11 [7,8] and YOLOv12 [9]. Nano-scale YOLO models are particularly attractive for onboard deployment because of their low parameter count and computational overhead. However, excessive lightweight design may weaken feature representation, especially in complex traffic scenes. Compared with general object detection tasks, traffic-scene detection involves large-scale variations between near and distant objects, frequent occlusion, and various imaging degradations. These factors increase the risk of missed detection for small and weakly textured objects, such as distant pedestrians, cyclists, traffic lights, and traffic signs. Recent studies [10,11] have also shown that task-oriented improvements based on traffic-scene characteristics are often more suitable for deployment than simply deepening or widening the network.

When lightweight YOLO models are applied to complex onboard traffic perception scenarios, three limitations become particularly important. First, the backbone is mainly constructed from stacked convolutional blocks, which are effective for local feature extraction but limited in modeling long-range contextual dependencies. As a result, small objects with weak texture, blurred boundaries, or low saliency may not be sufficiently represented. Introducing heavy attention modules can enhance global modeling, but it may also disturb the accuracy–efficiency balance required by lightweight detectors. Second, conventional feature pyramid structures usually rely on fixed upsampling and direct concatenation for cross-scale fusion. Such operations lack content-adaptive feature allocation and may not effectively balance high-level semantic information with shallow high-resolution details. Third, features directly fed into the detection head usually lack targeted spatial response calibration. This problem is more evident in the P3 branch, which is responsible for detecting small objects. Without further refinement, high-resolution features may contain redundant background responses and insufficient discriminative information for small-object localization.

To address these limitations, this study develops DER-YOLO, a lightweight stage-wise feature calibration network based on YOLO11n for small-object detection in complex traffic scenes. Instead of applying a heavy enhancement at a single location, DER-YOLO performs coordinated calibration across the backbone, neck, and pre-head stages. Following the detection pipeline, backbone-level global context calibration enhances the representation of weak-saliency small objects, neck-level adaptive cross-scale fusion preserves small-object details by balancing high-level semantics and shallow high-resolution features, and pre-head spatial refinement strengthens the high-resolution P3 feature for small-object localization before detection. This stage-wise design is intended to improve small-object representation while preserving the lightweight and real-time characteristics of YOLO11n. The main contributions of this work are summarized as follows:

1. A Decoupled Global Context C3k2 (DGC-C3k2) module is proposed for backbone feature calibration. By combining the local feature extraction structure of C3k2 with a lightweight global context enhancement unit, DGC-C3k2 strengthens semantic representation for weak and low-saliency traffic objects with limited additional complexity.
2. An ECA-guided Cross-scale Adaptive Fusion (ECAF) module is introduced into the P3 top-down fusion path. It combines dynamic upsampling, channel attention, and complementary weighting to adaptively balance high-level semantic information and shallow spatial details, thereby improving small-object feature fusion.
3. A Refined Large Selective Kernel (RLSK) module is designed as a pre-head spatial refinement component. It uses multi-branch depthwise convolutions with adaptive selection to calibrate high-resolution P3 features and enhance discriminative responses for small traffic objects.
4. Extensive experiments on KITTI and BDD100K demonstrate that DER-YOLO improves detection accuracy and small-object performance while maintaining lightweight real-time inference capability for onboard traffic perception applications.

## 2. Related Work

### 2.1. Lightweight Real-Time Detection and Small-Object Feature Enhancement

Since YOLOv1 [6], the YOLO series has evolved through continuous improvements in multi-scale prediction, feature aggregation, detection heads, and training strategies. YOLOv3 [12] introduced multi-scale prediction, YOLOv5 [13] integrated CSPNet [14] and PANet [15] structures, and YOLOv7 [16] explored efficient reparameterization strategies. More recent detectors, including YOLOv8 [17], YOLOv10 [18], YOLO11 [7,8], and YOLOv12 [9], further improved real-time detection through anchor-free heads, efficient backbone modules, end-to-end allocation strategies, and attention-oriented designs. RT-DETR [19] also demonstrated strong real-time detection capability by combining a hybrid encoder with a Transformer decoder. However, its parameter count and computational cost are usually higher than those of nano-scale YOLO models designed for onboard deployment. For onboard traffic perception systems, nano-scale detectors remain attractive because they provide a favorable balance between accuracy, model size, and inference speed. In addition to road-traffic scenarios, YOLO-based detection and tracking have also been applied to other autonomous platforms such as unmanned surface vehicles [20], reflecting the broad applicability of lightweight real-time detectors for autonomous perception.

Small-object detection in traffic and aerial scenes remains difficult because weak textures, blurred boundaries, occlusion, and scale variation often reduce feature discriminability [21,22]. Recent studies such as RE-YOLOv5 [23], LEAD-YOLO [24], YOLO-MXANet [25] and SOD-YOLOv8 [26] have shown that receptive-field enhancement, lightweight feature calibration, and attention-based fusion can improve small-object and traffic object detection in complex road-related scenarios. These studies indicate that lightweight detectors benefit from task-oriented feature representation for sensor-based perception, rather than simply increasing network depth or width.

Large-kernel and selective-kernel convolutions provide another way to enlarge receptive fields while retaining convolutional efficiency. RepLKNet [27] showed that large-kernel convolution can achieve competitive representation capability compared with visual Transformers. VAN [28] decomposed large-kernel attention into depthwise convolution, depthwise dilated convolution, and pointwise convolution for efficient receptive-field modeling. SKNet [29] introduced adaptive kernel selection through soft attention among branches with different receptive fields, while LSKNet [30] further developed large selective kernel modeling for object detection. Recent work on large separable kernel attention also suggests that efficient large-kernel design is useful for deployment-oriented vision models [31]. These methods provide useful inspiration for spatial response refinement. However, in lightweight traffic-scene detectors, large-kernel or selective-kernel mechanisms are often used in the backbone or neck, whereas their role as a pre-head refinement component for high-resolution small-object features in sensor-based traffic perception is less emphasized.

### 2.2. Global Context Modeling and Cross-Scale Feature Fusion

Effective traffic-scene detection requires both local details and global contextual information, especially for onboard vision-based traffic perception systems operating in complex road environments. SE-Net [32] introduced channel attention by using global average pooling and channel-wise reweighting. CBAM [33] further combined channel and spatial attention. Non-local Networks [34] modeled long-range dependencies through self-attention, but their computational cost is relatively high. GCNet [35] provided a more efficient alternative by simplifying non-local modeling and integrating global context aggregation with channel transformation. Coordinate attention [36] further introduced position-aware channel attention for lightweight networks. Recent detectors such as MDI-YOLO [37] and CMA-Net [38] also demonstrate that global-local feature interaction and attention-based feature enhancement can improve small-object detection. Nevertheless, directly inserting heavy attention modules into nano-scale detectors may disturb the balance between accuracy and efficiency required for onboard deployment.

Cross-scale feature fusion is another key factor for small-object detection. FPN [39] established a top-down feature fusion paradigm, and PANet [15] added bottom-up path aggregation. BiFPN [40] introduced learnable bidirectional fusion weights, while NAS-FPN [41] searched for better fusion topologies. In addition to fusion topology, upsampling quality also affects small-object representation. Fixed interpolation is simple but lacks content awareness, which may smooth object boundaries and weaken high-resolution details. CARAFE [42] performed content-aware feature reassembly by generating adaptive kernels, and DySample [43] achieved dynamic upsampling through learnable point sampling with low computational overhead. These methods improve individual aspects of multi-scale representation, but many lightweight detectors still rely on fixed upsampling and direct concatenation in the neck, which may be insufficient for preserving weak small-object cues in traffic-scene imaging.

Overall, previous studies have provided effective components for global context modeling, adaptive upsampling, channel attention, and receptive-field enlargement. However, for resource-constrained onboard traffic perception, it remains important to determine how these functions should be positioned along the detection pipeline. This work therefore focuses on stage-wise feature calibration in YOLO11n: global context calibration in the backbone, adaptive cross-scale fusion in the P3 neck path, and pre-head spatial response refinement before the P3 detection head. This design aims to improve small-object representation in complex traffic scenes while preserving lightweight real-time inference for onboard traffic perception applications.

## 3. Methods

This study uses YOLO11n as the baseline detector and develops DER-YOLO for lightweight small-object detection in complex traffic scenes. DER-YOLO addresses three main limitations of lightweight detectors: insufficient backbone context representation, inadequate cross-scale feature fusion, and limited pre-head feature calibration. To tackle these issues, DGC-C3k2, ECAF, and RLSK modules are introduced into the backbone, neck, and pre-head stages, respectively, forming a stage-wise feature calibration framework that enhances small-object representation while maintaining lightweight real-time performance.

### 3.1. Overall Structure

**Figure 1:**
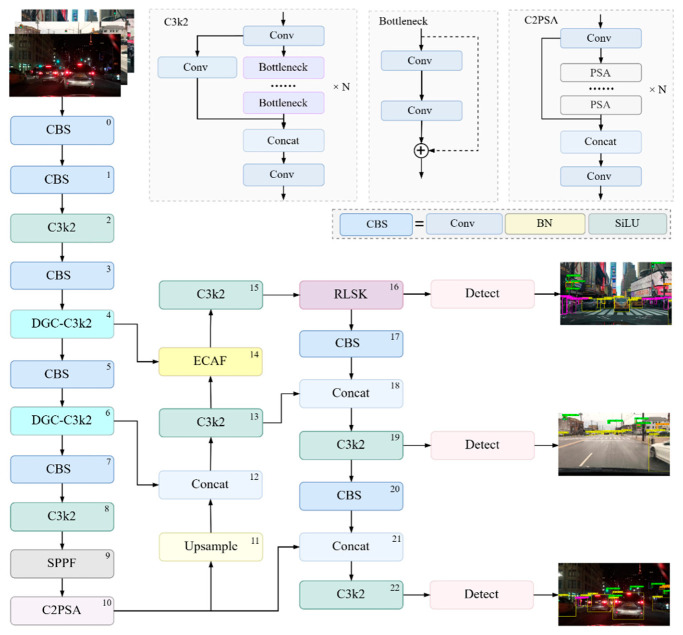
Overall architecture of DER-YOLO.

The overall architecture of DER-YOLO is shown in Figure 1. In the backbone, the C3k2 modules at the P3 and P4 stages of YOLO11n are replaced with DGC-C3k2 modules, which preserve local feature extraction and append DGC-Net for global context calibration. In the neck, an ECAF module is introduced into the P3 top-down fusion path. It replaces fixed upsampling and direct concatenation with DySample-based dynamic upsampling, ECA [44] channel attention, and complementary Softmax weighting, enabling adaptive fusion of high-level semantics and shallow high-resolution details. In the pre-head stage, an RLSK module is inserted after the P3 C3k2 feature encoding block and before the P3 detection head. It refines high-resolution spatial responses through multi-branch depthwise convolutions and lightweight adaptive selection, thereby enhancing small-object-related feature responses before prediction.

### 3.2. DGC-C3k2 Module

**Figure 2:**
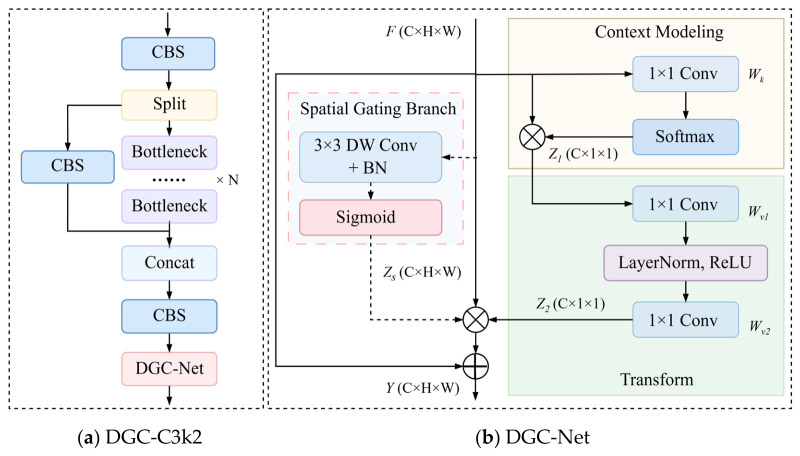
Architecture of the DGC-C3k2 module. (**a**) Overall structure of DGC-C3k2; (**b**) internal structure of DGC-Net. The dashed Spatial Gating Branch represents a candidate spatial modulation path evaluated during module design, which is not included in the final DGC-Net structure because it did not improve performance in the ablation study.

In traffic scenes, small objects such as distant pedestrians, cyclists, and traffic signs often have weak textures, blurred boundaries, and low saliency. These characteristics require the backbone to capture not only local details but also broader contextual information. Although C3k2 in YOLO11n is efficient for lightweight feature extraction, its convolution-based structure mainly models local receptive fields and has limited ability to capture long-range dependencies. Therefore, DGC-C3k2 is designed by appending a lightweight global context enhancement unit, termed DGC-Net, to the original C3k2 structure. As shown in Figure 2a, DGC-C3k2 first preserves the dual-branch local feature extraction of C3k2 and then performs global context calibration on the fused feature.

Let the input feature map of the DGC-C3k2 module be denoted as $X\in R^{C\times H\times W}$, where *C*, *H* and *W* represent the number of channels, height, and width, respectively. After an initial projection by a CBS module, the input feature is split along the channel dimension into two branch features, denoted as $X_{1}$ and $X_{2}$. Specifically, $X_{1}$ is fed into a CBS-based direct path to preserve shallow detailed information, while $X_{2}$ is passed through the main branch composed of N cascaded Bottleneck blocks to extract deep semantic features. The two branch outputs are then concatenated along the channel dimension and integrated by another CBS module to obtain an intermediate feature F. The feature F is then fed into DGC-Net for global context modeling. The above process can be expressed as follows:

(1) $$F=\mathrm{CBS}\left(\mathrm{Concat}\left(\mathrm{CBS}\left(X_{1}\right),{\mathrm{Bottleneck}}^{N}\left(X_{2}\right)\right)\right),$$

(2) Y=DGCNet(F),

As shown in Figure 2b, DGC-Net consists of two main components: Context Modeling and Transform. The Context Modeling component aggregates global contextual information along the spatial dimension using learnable spatial weights, while the Transform component maps the aggregated global descriptor into a channel enhancement vector.

In the Context Modeling component, the input feature F is first projected by a 1 × 1 convolution to generate a single-channel spatial response map. Softmax normalization is then applied along the spatial dimension to obtain the spatial weight $\alpha_{i}$ for the i-th location:

(3) $$\alpha_{i}=\frac{\exp(s_{i})}{\sum_{j=1}^{HW}\exp(s_{j})},$$

where $s_{i}$ denotes the spatial response value at the corresponding location. Subsequently, these spatial weights are used to perform a weighted aggregation of the channel vectors over all spatial locations, yielding the global context vector $Z_{1}\in R^{C\times 1\times 1}$:

(4) $$Z_{1}=\sum_{i=1}^{HW}\alpha_{i}F_{i},$$

Different from global average pooling, this weighted aggregation allows DGC-Net to emphasize spatial regions that contribute more to object representation.

In the Transform branch, the global context vector $Z_{1}$ is further passed through two successive 1 × 1 convolution layers parameterized by $W_{v1}$ and $W_{v2}$, respectively. Combined with LayerNorm and the ReLU activation function for nonlinear transformation, this process yields the channel enhancement vector $Z_{2}$, which can be expressed as follows:

(5) $$Z_{2}=W_{v2}\delta \left(\mathrm{LN}\left(W_{v1}Z_{1}\right)\right),$$

where LN(·) denotes the LayerNorm operation, and δ(·) represents the ReLU activation function. The role of this branch is to further map the global contextual information into the channel dimension, thereby generating selective channel enhancement responses and enhancing the representation capability of channels relevant to the detection targets.

During the module design, a Spatial Gating Branch was also explored as a candidate spatial enhancement component. Specifically, this branch employs a 3 × 3 depthwise convolution, batch normalization, and a Sigmoid activation function to generate a spatial gating response $Z_{s}\in R^{C\times H\times W}$, which is used to perform element-wise spatial modulation on the input feature, thereby further emphasizing potential target regions while suppressing irrelevant background responses. However, the ablation results indicate that this spatial gating branch does not bring additional performance gains and instead slightly degrades the detection accuracy. Therefore, this branch is removed in the final model, and DGC-Net retains only the Context Modeling and Transform components.

Based on the final adopted structure, the channel-enhancement vector $Z_{2}$ is applied to the input feature *F* as a residual multiplicative channel gate, rather than being added directly. The output of DGC-Net is therefore obtained as:

(6) $$Y=F+F⊙Z_{2},$$

where ⊙ denotes element-wise multiplication broadcast over the spatial dimensions, with $Z_{2}\in R^{C\times 1\times 1}$. Owing to the residual term, this gate emphasizes informative channels according to their global-context relevance while preserving the original feature responses.

Through this design, DGC-C3k2 combines the local feature extraction capability of C3k2 with lightweight global context calibration. The “local encoding followed by global modulation” strategy enhances small-object-related semantic representation while avoiding the repeated insertion of attention modules inside the backbone.

Compared with SE and ECA, DGC-Net uses attention-based spatial aggregation rather than global average pooling to obtain the context descriptor. Compared with GCNet, DGC-Net uses the transformed context to modulate feature channels before combining them with the original feature, rather than adding it directly as a context residual.

### 3.3. ECAF Module

**Figure 3:**
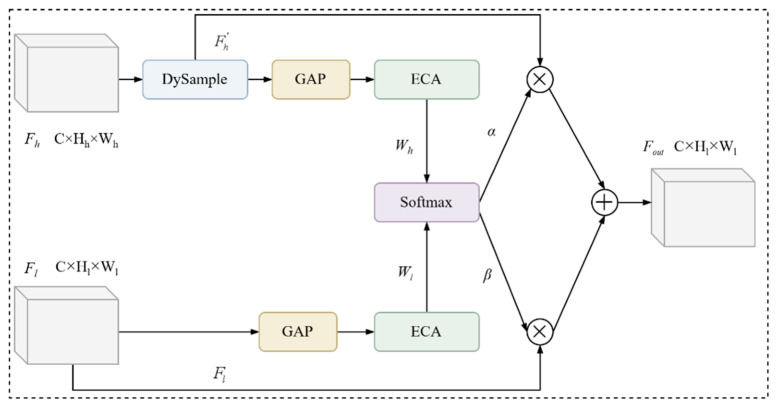
Architecture of the ECAF module.

The conventional feature pyramid structure has two limitations for small-object detection. First, fixed upsampling lacks content awareness and may smooth the edges and textures of small objects. Second, direct concatenation treats high-level semantic features and shallow detailed features with equal importance, without adaptive contribution allocation. To address these limitations, an ECAF module is introduced into the P3 top-down fusion path, as shown in Figure 3. ECAF first uses DySample to dynamically upsample the high-level low-resolution feature. It then applies ECA to both the reconstructed high-level feature and the shallow high-resolution feature. Finally, complementary channel-level fusion weights are generated by Softmax normalization to adaptively balance semantic and detailed information.

The feature map generated by the high-level branch is denoted as $F_{h}\in R^{C\times H_{h}\times W_{h}}$, while the shallow feature from the lateral connection is denoted as $F_{l}\in R^{C\times H_{l}\times W_{l}}$. The high-level feature contains stronger semantic information but has lower spatial resolution, whereas the shallow feature preserves more detailed spatial information. ECAF first applies DySample to reconstruct the high-level feature to the same resolution as $F_{l}$:

(7) $$F_{h}^{\prime }=\mathrm{DySample}\left(F_{h}\right),$$

Then, ECA is applied to $F_{h}^{\prime }$ and $F_{l}$ to generate the channel response weights $W_{h}$ and $W_{l}$. For an input feature $X\in R^{C\times H\times W}$, global average pooling, denoted by GAP(⋅), produces a channel descriptor $z=GAP(X)\in R^{C\times 1\times 1}$, whose c-th component is:

(8) $$z_{c}=\frac{1}{HW}\sum_{i=1}^{H}\sum_{j=1}^{W}X_{c}\left(i,j\right),$$

The channel weights are then obtained by a local one-dimensional convolution followed by a Sigmoid activation:

(9) $$W\left(X\right)=\sigma \left(\mathrm{Conv}1D\left(\mathrm{GAP}\left(X\right)\right)\right),$$

Accordingly, the channel responses of the two branches are:

(10) $$W_{h}=W\left(F_{h}^{\prime }\right),\;W_{l}=W\left(F_{l}\right),$$

To adaptively allocate the contribution of the two branches, Softmax normalization is applied channel-wise to generate complementary fusion weights $\alpha ,\beta \in R^{C\times 1\times 1}$:

(11) $$\alpha_{c}=\frac{\exp\left(W_{h,c}\right)}{\exp\left(W_{h,c}\right)+\exp\left(W_{l,c}\right)},\;\beta_{c}=\frac{\exp\left(W_{l,c}\right)}{\exp\left(W_{h,c}\right)+\exp\left(W_{l,c}\right)},$$

where $\alpha_{c}+\beta_{c}=1$ for each channel c. The fused output is obtained by channel-wise weighted summation:

(12) $$F_{\mathrm{out}}=\alpha ⊙F_{h}^{\prime }+\beta ⊙F_{l},$$

By combining dynamic upsampling with channel-wise complementary weighting, ECAF adaptively integrates high-level semantic information and shallow high-resolution details in the P3 branch. Rather than concatenating the two branches, it performs channel-wise weighted summation, preserving the channel dimension and avoiding the extra cost of subsequent channel-reduction convolution.

Unlike ECA, which independently recalibrates a single feature map, ECAF applies channel attention to both branches and converts their responses into complementary fusion weights through channel-wise Softmax normalization. DySample is used only to reconstruct the high-level feature, whereas the cross-scale fusion is completed by weighted summation. This design enables explicit cross-branch contribution allocation with limited additional complexity.

### 3.4. RLSK Module

**Figure 4:**
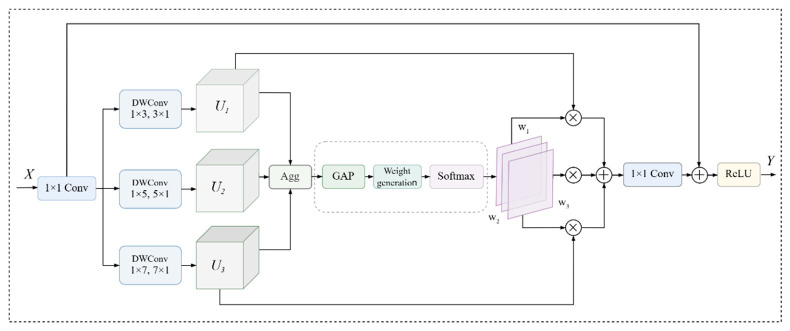
Architecture of the RLSK module.

In traffic scenes, object scales vary significantly. Distant pedestrians, cyclists, and traffic signs require fine local details, whereas larger nearby vehicles benefit from broader contextual responses. Fixed receptive-field convolutions are limited in adapting to such scale variation. To refine the high-resolution P3 feature before prediction, an RLSK module is inserted after the P3 C3k2 feature encoding block and before the P3 detection head, as shown in Figure 4. After a 1 × 1 pre-mixing convolution, RLSK uses three parallel strip depthwise convolution branches with equivalent kernel sizes of 3 × 3, 5 × 5, and 7 × 7 to capture spatial responses at different granularities, where each branch decomposes a *k* × *k* depthwise convolution into a 1 × *k* convolution followed by a *k* × 1 convolution to enlarge the receptive field with fewer parameters. A lightweight adaptive selection mechanism is then used to assign branch weights and fuse these multi-granularity features, improving the discriminative representation of small-object regions with limited additional computational cost.

Let the input feature map be denoted as $X\in R^{C\times H\times W}$, where C, H, and W represent the number of channels, height, and width, respectively. RLSK first applies a 1 × 1 pre-mixing convolution followed by batch normalization and a SiLU activation to obtain a mixed feature:

(13) $$X^{\prime }=\sigma \left(\mathrm{BN}\left({\mathrm{Conv}}_{1\times 1}\left(X\right)\right)\right),$$

where σ(⋅) denotes the SiLU activation. Three parallel strip depthwise convolution branches then extract spatial features at different granularities:

(14) $$U_{1}=f_{3}\left(X^{\prime }\right),\;U_{2}=f_{5}\left(X^{\prime }\right),\;U_{3}=f_{7}\left(X^{\prime }\right),$$

where $f_{k}(\cdot )$ denotes a strip depthwise convolution unit with equivalent kernel size *k* × *k*, implemented as a 1 × *k* depthwise convolution followed by a *k* × 1 depthwise convolution, each with batch normalization and SiLU activation. The three branch features correspond to local, medium-range, and larger receptive-field spatial responses. They are first aggregated to obtain a global descriptive feature:

(15) $$U=U_{1}+U_{2}+U_{3}$$

Then, global average pooling is applied to *U* to generate a compact channel descriptor $z\in R^{C\times 1\times 1}$:

(16) $$z=\mathrm{GAP}\left(U\right),$$

The descriptor is mapped by a lightweight weight generation function g(⋅), and Softmax is applied along the branch dimension to obtain adaptive branch weights:

(17) $$\left[w_{1},w_{2},w_{3}\right]=\mathrm{Softmax}\left(g\left(z\right)\right),$$

where $w_{i}\in R^{C\times 1\times 1}$ denotes the channel-wise weight of the i-th branch, and $w_{1}+w_{2}+w_{3}=1$ for each channel. The three branch features are then selectively fused as:

(18) $$F_{ref}=w_{1}⊙U_{1}+w_{2}⊙U_{2}+w_{3}⊙U_{3},$$

where ⊙ denotes element-wise multiplication. Finally, a 1 × 1 convolution and residual connection are used to obtain the output feature:

(19) $$Y=\delta \left(f_{1\times 1}\left(F_{ref}\right)+X\right),$$

where $f_{1\times 1}(\cdot )$ denotes the 1 × 1 convolution and $\delta \left(\cdot \right)$ represents the ReLU activation.

RLSK differs from existing selective-kernel designs mainly in its lightweight implementation and placement. Unlike SKNet and LSKNet, which employ selective kernels for backbone feature extraction and receptive-field modeling, RLSK uses strip depthwise convolution branches with residual detail preservation as a pre-head refinement module. It is applied only to the high-resolution P3 feature before detection, enabling targeted spatial-response calibration for small objects with limited additional cost.

## 4. Experimental Results and Analysis

### 4.1. Datasets

Experiments were conducted on two public autonomous driving datasets, KITTI [45] and BDD100K [46]. KITTI is a standard benchmark for autonomous driving perception and includes common challenges such as illumination changes, occlusion, and small distant objects. Following common practice, “Truck” and “Van” were merged into the “car” category, and “Person sitting” was merged into “pedestrian”, resulting in three final categories: “car”, “pedestrian”, and “cyclist”. The 7481 KITTI images were divided into 5984 training images and 1497 validation images using a fixed 8:2 split.

BDD100K contains more complex traffic scenes, with denser object distributions and larger scale variations, making it suitable for evaluating detection performance in urban driving environments. Because the original annotations contain many categories, the labels were reorganized in this study to better match the task setting and to reduce the influence of long-tail categories and overly fine-grained class definitions. Specifically, four categories were used: “vehicle”, “pedestrian”, “traffic sign”, and “traffic light”. All vehicle-related classes were merged into “vehicle”, and all pedestrian-related classes were merged into “pedestrian”, while “traffic sign” and “traffic light” were kept separate because of their importance in autonomous driving perception. In this study, the official BDD100K training and validation sets were used. The training set was used for model optimization, while the validation set was used for model selection and performance evaluation.

**Figure 5:**
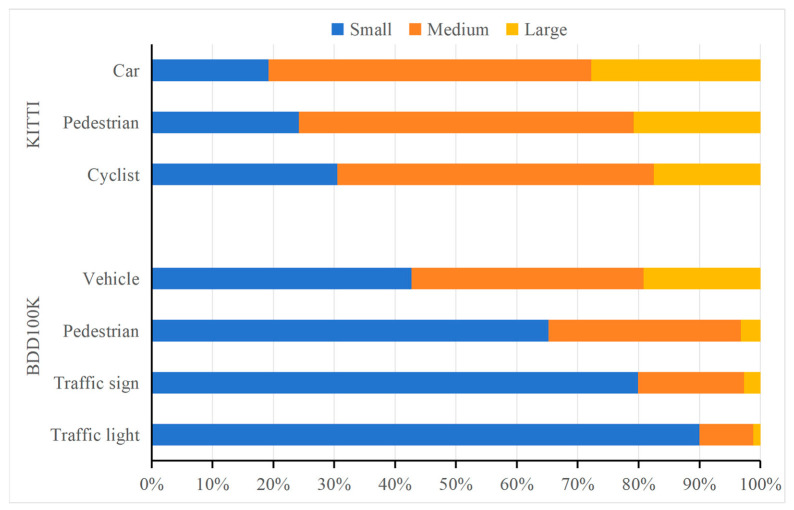
Proportional scale distribution of object instances in the reorganized KITTI and BDD100K datasets. Statistics are calculated over the complete training and validation splits of KITTI and BDD100K. Object scales are determined using the COCO criterion based on the bounding-box area in the original image coordinates before resizing: small, area < 32^2^ pixels; medium, 32^2^ ≤ area < 96^2^ pixels; and large, area ≥ 96^2^ pixels.

Both datasets contain traffic objects with large-scale variations, including many distant and low-resolution instances, making them suitable for evaluating small-object detection under challenging traffic-scene conditions. Accordingly, Figure 5 reports the category-wise distribution of small, medium, and large object instances in the reorganized KITTI and BDD100K datasets.

While Figure 5 illustrates the proportional scale composition of each category, Table 1 provides the corresponding absolute instance counts and per-category totals, thereby offering clearer sample-size context for the subsequent category-wise small-object analysis.

### 4.2. Experimental Setup and Training Settings

The hardware and software environment, together with the main training hyperparameter settings, are summarized in Table 2. Unless otherwise specified, all experiments were conducted under the same configuration, with the input image size fixed at 640 × 640. Data augmentation included Mosaic, HSV augmentation, random scaling, and horizontal flipping. To ensure a fair comparison, all models in the ablation and comparison experiments were trained from scratch under identical settings without pretrained weights (Section 4.4.5 separately examines the effect of COCO-pretrained initialization). For inference-speed evaluation, FPS was measured on the same platform using a batch size of 1 and without TensorRT acceleration after several warm-up iterations.

### 4.3. Evaluation Metrics

This study uses standard object detection metrics to evaluate model performance, including precision (*P*), recall (*R*), mAP@0.5, mAP@0.5:0.95, parameters (Params), computational cost (GFLOPs), and inference speed (FPS). Precision and recall are defined as:

(20) $$P=\frac{\mathrm{TP}}{\mathrm{TP}\;+\;\mathrm{FP}},$$

(21) $$R=\frac{\mathrm{TP}}{\mathrm{TP}+\mathrm{FN}},$$

where TP, FP, and FN denote true positives, false positives, and false negatives, respectively. The average precision (AP) of a single class is computed from the precision-recall curve:

(22) $$\mathrm{AP}=\int_{0}^{1}P\left(r\right)dr,$$

The mean average precision (mAP) is defined as the mean value of AP over all categories:

(23) $$\mathrm{mAP}=\frac{1}{N}\sum_{i=1}^{N}{\mathrm{AP}}_{i},$$

where *N* is the number of object categories. mAP@0.5 is computed at an intersection-over-union (IoU) threshold of 0.5, whereas mAP@0.5:0.95 is averaged over IoU thresholds from 0.5 to 0.95 with a step size of 0.05. Params, GFLOPs, and FPS are used to evaluate model complexity and inference efficiency.

### 4.4. Ablation and Analysis Experiments

All ablation experiments were conducted on the KITTI dataset using the same data split and training settings to ensure fair comparison.

#### 4.4.1. DGC-C3k2 Ablation and Comparison

We first analyze the internal design of DGC-Net (Table 3) and then compare DGC-C3k2 with existing attention and context modules (Table 4).

As shown in Table 3, retaining Context Modeling alone reduces mAP@0.5 by 1.06 points, since context aggregation without channel-selective mapping cannot enhance features effectively. Adding Transform (C + T) raises mAP@0.5 to 85.32% and mAP@0.5:0.95 to 58.53%, showing that channel remapping improves the use of global context. Further adding the Spatial Gating Branch (Mul or Res) brings no accuracy gain but slightly increases cost. The final DGC-Net therefore adopts the C + T structure.

Table 4 compares DGC-C3k2 with four representative attention and context modules, namely SE, CBAM, CA, and GCNet, which are inserted at the same backbone positions under comparable computational budgets. GCNet performs best among the competing modules, whereas CBAM performs worse than the baseline. DGC-C3k2 achieves the highest accuracy among all variants and slightly surpasses GCNet, although it introduces a modest increase in parameters and computational cost. Since all modules are evaluated at the same backbone positions under comparable budgets, DGC-C3k2 attains accuracy on par with the strongest competing module, indicating that the proposed attention-based context aggregation and residual multiplicative channel gating offer an effective context-calibration design for lightweight backbones, rather than a gain driven merely by added capacity.

#### 4.4.2. ECAF Ablation and Comparison

Table 5 compares upsampling operators and fusion strategies for the cross-scale fusion. Among the upsamplers, the learnable CARAFE and DySample both outperform fixed nearest-neighbor interpolation, with DySample performing best, confirming the benefit of content-aware upsampling. Building on DySample, ECAF replaces concatenation-and-convolution fusion with a lightweight ECA-weighted adaptive fusion. This yields the highest mAP@0.5 while using the fewest parameters and the lowest computation among all variants, since ECAF avoids the channel-reduction convolution that concatenation requires. The DySample+concat variant attains a slightly higher mAP@0.5:0.95, but at roughly 21% more GFLOPs and additional parameters. For real-time onboard traffic detection, where computational budget is the binding constraint, ECAF’s ability to deliver the best mAP@0.5 at the lowest cost makes it the more favorable choice.

#### 4.4.3. RLSK Ablation and Placement Analysis

We examine the receptive-field branch design of RLSK in Table 6 and its placement across feature scales in Table 7. These experiments assess whether performance gains arise from multi-granularity branch selection and from targeted refinement at the high-resolution small-object scale.

As shown in Table 6, the 3 × 3 branch alone slightly improves mAP@0.5 and mAP@0.5:0.95 over the baseline, indicating that local spatial refinement is beneficial but limited. Adding the 5 × 5 branch further improves performance, suggesting that a moderately larger receptive field helps capture richer spatial context. The combination of 3 × 3, 5 × 5, and 7 × 7 branches achieves the best results, with mAP@0.5 and mAP@0.5:0.95 reaching 85.21% and 58.56%, respectively. Meanwhile, the parameter count increases by only 0.054M compared with the baseline. Therefore, the three-branch configuration is adopted in the final RLSK module.

Table 7 compares RLSK placement across pyramid levels. All variants outperform the baseline, and P3 gives the best single-level result, consistent with RLSK being most effective at P3 level for small objects. Although P3 + P4 + P5 reaches the highest accuracy, its gain over P3-only is marginal while parameters and GFLOPs rise noticeably. RLSK is therefore placed only at P3 for a better accuracy–efficiency trade-off.

#### 4.4.4. Overall Module Ablation

To evaluate the individual and combined effects of DGC-C3k2, ECAF, and RLSK, overall module ablation experiments were conducted on the KITTI dataset. Model ① denotes the YOLO11n baseline, and subsequent models progressively introduce different module combinations. The results are summarized in Table 8.

This study evaluates the individual contributions of DGC-C3k2, ECAF, and RLSK. Compared with baseline model ①, models ②, ③, and ④ introduce DGC-C3k2, ECAF, and RLSK, respectively. Model ② improves mAP@0.5 to 85.32%, while model ③ achieves 85.55% mAP@0.5 and 76.78% recall, demonstrating the effectiveness of cross-scale fusion. Model ④ mainly improves mAP@0.5:0.95 from 58.11% to 58.56%, suggesting a potential benefit for localization quality.

Model ⑤ combines DGC-C3k2 and ECAF and achieves 86.33% mAP@0.5 and 59.84% mAP@0.5:0.95, outperforming the single-module variants. With the further addition of RLSK, model ⑥ achieves the best performance of 86.95% mAP@0.5 and 60.89% mAP@0.5:0.95, improving by 2.07 and 2.78 percentage points over model ①. Although complexity increases, model ⑥ still runs at 111.4 FPS, showing a favorable accuracy-efficiency trade-off.

#### 4.4.5. Effect of Pretrained Initialization

In addition, Table 9 presents the results obtained with scratch training and official COCO-pretrained initialization on KITTI. With pretrained weights, YOLO11n improved from 84.88% mAP@0.5 and 58.11% mAP@0.5:0.95 to 85.91% and 59.38%, respectively. DER-YOLO increased from 86.95% mAP@0.5 and 60.89% mAP@0.5:0.95 to 87.72% and 61.94%, respectively. DER-YOLO remained superior to YOLO11n under both training strategies.

### 4.5. Detection Performance Analysis

#### 4.5.1. Class-Wise Performance Analysis

**Figure 6:**
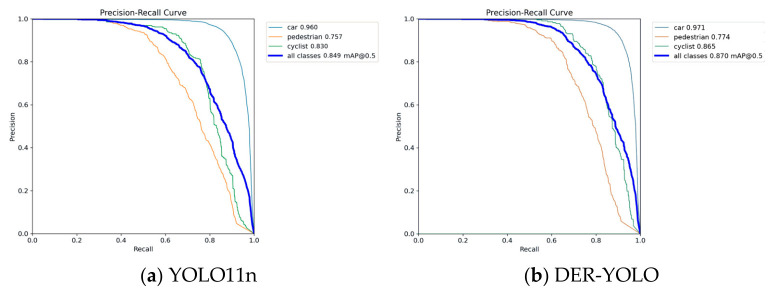
P-R curves for each class of YOLO11n and DER-YOLO on KITTI.

**Figure 7:**
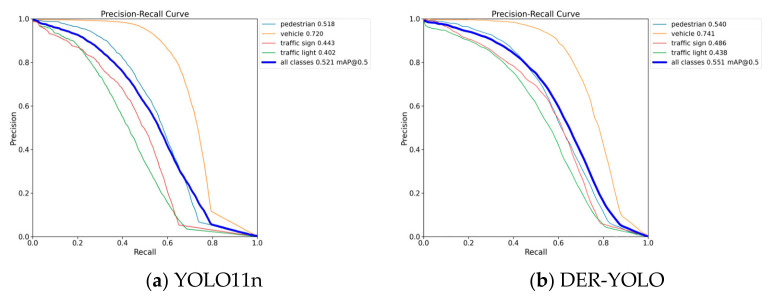
P-R curves for each class of YOLO11n and DER-YOLO on BDD100K.

Figure 6 and Figure 7 illustrate the P-R curves of YOLO11n and the proposed DER-YOLO on the KITTI and BDD100K datasets. Under the mAP@0.5 evaluation metric, DER-YOLO consistently outperforms YOLO11n on both datasets. Specifically, the all-class mAP@0.5 on KITTI increases from 0.849 to 0.870, while that on BDD100K improves from 0.521 to 0.551. For the KITTI dataset, DER-YOLO improves the AP values of car, pedestrian, and cyclist from 0.960, 0.757, and 0.830 to 0.971, 0.774, and 0.865, among which the cyclist category obtains the largest improvement. For the BDD100K dataset, the AP values of vehicle, pedestrian, traffic sign, and traffic light increase from 0.720, 0.518, 0.443, and 0.402 to 0.741, 0.540, 0.486, and 0.438. The improvements in traffic sign and traffic light are relatively more evident, indicating that DER-YOLO is more effective in detecting small and densely distributed traffic objects. Overall, these results verify the effectiveness of the proposed method in enhancing detection accuracy and robustness across different traffic-scene datasets.

#### 4.5.2. Scale-Wise Performance Analysis

To further verify the small-object-oriented design of DER-YOLO, this section analyzes detection performance from the perspective of target scale. By separately evaluating AP for small, medium, and large objects, the scale-wise results provide a more detailed assessment of the model’s capability in handling small-scale targets.

According to the COCO evaluation protocol [47], object instances are divided into small, medium, and large objects, and the AP values for each scale group are reported separately. The official COCO evaluation toolkit, pycocotools, was used for this assessment. Therefore, both the scale-wise AP/AR results in Table 10 and the category-wise small-object AP results in Table 11 are reported under the COCO protocol and may differ slightly from the mAP values obtained using the Ultralytics evaluation pipeline.

The results in Table 10 show that DER-YOLO outperforms the baseline model across all scale groups on both datasets, with the largest gains observed for small objects. On KITTI, AP@0.5:0.95 for small objects improves by 2.3 percentage points, while the gain for large objects is only 0.8 percentage points. On BDD100K, the small-object AP increases by 2.6 percentage points, compared with 0.5 percentage points for large objects. In addition, the improvement in AP@0.75 is greater than that in AP@0.5, indicating that the proposed modules not only enhance detection capability but also improve localization accuracy. The AR results show a similar trend. These findings suggest that global context enhancement in DGC-C3k2, cross-scale adaptive fusion in ECAF, and spatial response calibration in RLSK jointly contribute to more effective small-object detection in complex traffic scenes.

Although the scale-wise results confirm the overall effectiveness of DER-YOLO on small objects, the performance gains may still vary across categories. Therefore, to further examine whether the overall small-object improvement masks inter-category differences, category-wise small-object AP values were additionally evaluated, as shown in Table 11.

As shown in Table 11, DER-YOLO consistently improves the category-wise small-object AP on both datasets. On KITTI, the largest improvement is observed for Cyclist, followed by Pedestrian and Car. On BDD100K, DER-YOLO also shows clear improvements for small-scale objects, especially Traffic sign and Traffic light, which contain a high proportion of small instances. These results further demonstrate that the small-object improvements of DER-YOLO are not limited to the overall scale-level AP but are consistently reflected across different traffic-scene categories.

### 4.6. Comparison Experiments

To evaluate the performance of DER-YOLO more comprehensively, we compared it with several commonly used object detection methods on the KITTI and BDD100K datasets. All methods were trained and evaluated using the same dataset partition, input resolution of 640 × 640, and hardware platform.

#### 4.6.1. Comparison Results on KITTI

As shown in Table 12, DER-YOLO achieves 86.95% mAP@0.5 and 60.89% mAP@0.5:0.95 on KITTI, improving by 2.07 and 2.78 percentage points over the YOLO11n baseline. It also outperforms NanoDet-Plus-m [48], YOLOv8n, YOLOv10n, and YOLOv12n in both accuracy metrics. Although YOLO11s and HIC-YOLOv5 [49] achieve higher accuracy, DER-YOLO provides a more favorable efficiency trade-off with substantially fewer parameters and lower computational cost. Compared with RT-DETR-r18, DER-YOLO improves both mAP@0.5 and mAP@0.5:0.95 while using only 13.8% of the parameters and 13.0% of the GFLOPs. These results demonstrate that DER-YOLO effectively improves detection accuracy while maintaining a lightweight architecture and real-time inference capability.

#### 4.6.2. Comparison Results on BDD100K

Table 13 shows that BDD100K is more demanding than KITTI in data scale, target categories, and scene diversity. Here, DER-YOLO reaches 55.05% mAP@0.5 and 29.21% mAP@0.5:0.95, exceeding the YOLO11n baseline by 3.00 and 2.70 percentage points, respectively. It likewise surpasses NanoDet-Plus-m, YOLOv8n, YOLOv10n, YOLOv12n, and RT-DETR-r18 on both accuracy metrics. YOLO11s and HIC-YOLOv5 attain higher accuracy on both metrics; however, DER-YOLO offers a more favorable accuracy–efficiency trade-off, with far fewer parameters and lower computational cost. Compared with the larger YOLO11s of the same series, DER-YOLO attains comparable accuracy while using only 29.1% of the parameters and 34.3% of the GFLOPs and running about 1.2 times faster. Against HIC-YOLOv5 specifically, DER-YOLO attains nearly identical mAP@0.5:0.95 while using just 29.5% of the parameters and 23.9% of the GFLOPs and running about 1.5 times faster. These findings confirm that the stage-wise feature calibration strategy generalizes beyond KITTI to the larger and more complex BDD100K, demonstrating strong cross-dataset adaptability.

The results on KITTI and BDD100K demonstrate the overall effectiveness of DER-YOLO in terms of detection accuracy, model complexity, and inference speed. To confirm that the gain of DER-YOLO over the baseline is not caused by random fluctuations, both models were trained three times with different seeds. The three-run results are consistent with the single-run values, and the improvement consistently exceeds the run-to-run variation.

### 4.7. Visualization Analysis

Visual results are further provided to analyze the detection behavior of DER-YOLO, including loss function curves, object detection results, and gradient-weighted class activation mapping (Grad-CAM) [50] visualizations. The loss curves are used to compare the training convergence of YOLO11n and DER-YOLO, the detection results illustrate their performance in representative traffic scenes, and the Grad-CAM visualizations provide an intuitive interpretation of the target-related response regions learned by the models.

#### 4.7.1. Loss Function Curves

**Figure 8:**
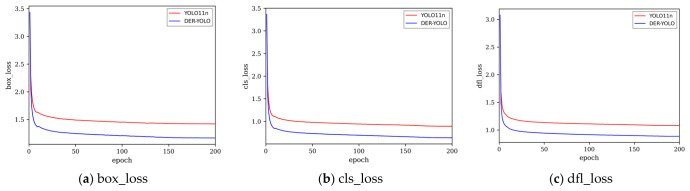
Training loss curves of YOLO11n and DER-YOLO on BDD100K: (**a**) box_loss; (**b**) cls_loss; (**c**) dfl_loss.

To compare training convergence, the loss trends of YOLO11n and DER-YOLO were recorded on the BDD100K dataset. As shown in Figure 8, DER-YOLO reaches lower convergence values than YOLO11n in terms of box_loss, cls_loss, and dfl_loss, indicating improved optimization behavior in bounding box regression, classification, and distribution focal loss. All three curves show a stable downward trend without obvious oscillation, suggesting that DER-YOLO maintains stable training convergence on BDD100K.

#### 4.7.2. Object Detection Results

**Figure 9:**
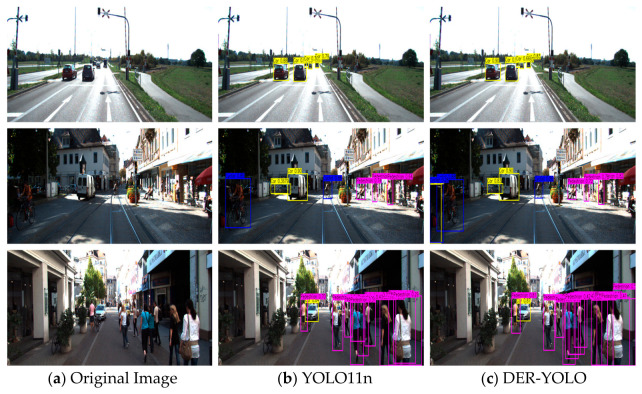
Detection result comparison on KITTI: (**a**) original image; (**b**) YOLO11n; (**c**) DER-YOLO.

**Figure 10:**
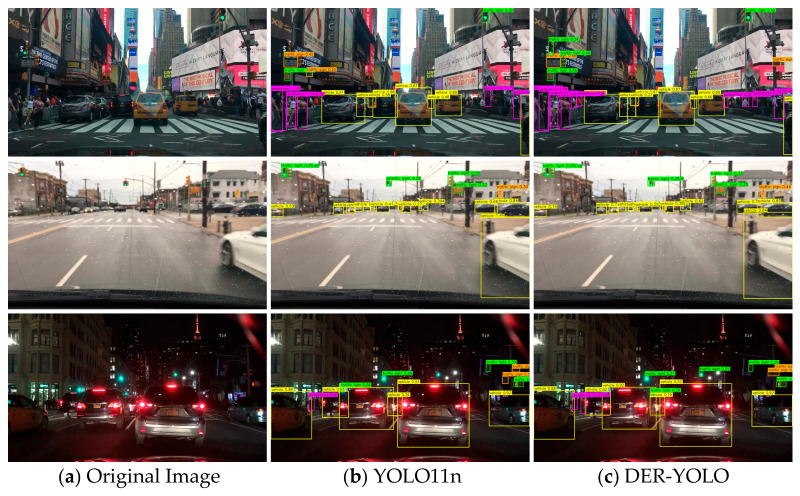
Detection result comparison on BDD100K: (**a**) original image; (**b**) YOLO11n; (**c**) DER-YOLO.

To further evaluate the robustness and generalization capability of DER-YOLO in autonomous driving scenarios, representative complex traffic scenes were selected from the KITTI and BDD100K datasets for qualitative visual comparison. The KITTI examples include mixed scenes with pedestrians and cyclists, open-road scenes containing distant vehicles, and intersection scenes with multiple interacting targets. The BDD100K examples include complex urban scenes, rainy conditions, and nighttime environments. The comparative results are shown in Figure 9 and Figure 10.

Particular attention is given to distant or low-resolution traffic objects, because they often appear as small-scale instances in onboard traffic-scene images. As shown in Figure 9 and Figure 10, DER-YOLO shows better detection performance than the YOLO11n baseline across representative KITTI and BDD100K scenarios. On KITTI, DER-YOLO achieves higher confidence for distant small vehicles and recalls some targets missed by the baseline. In mixed urban scenes and dense pedestrian scenarios, it provides more complete detection of pedestrians and cyclists, with fewer missed detections and tighter bounding box localization. On BDD100K, DER-YOLO detects dense vehicles and small traffic signs more completely in complex urban scenes. Under degraded conditions such as rainy weather and nighttime environments, it also maintains stable responses to distant targets, pedestrians, and traffic signals. These qualitative results are consistent with the quantitative findings and further suggest that DGC-C3k2, ECAF, and RLSK jointly improve the detection of small and low-saliency traffic objects in complex scenes.

#### 4.7.3. Grad-CAM Results

Grad-CAM [50] is a visualization technique for interpreting deep learning models. It analyzes the gradients of the target class with respect to convolutional feature maps and generates a heatmap that highlights the regions contributing to the model prediction. This provides an intuitive way to observe the target-related response regions learned by DER-YOLO. For both YOLO11n and DER-YOLO, Grad-CAM was applied to the high-resolution P3 feature immediately before the P3 detection head. For DER-YOLO, this feature corresponds to the output of the RLSK module.

For each image, the correctly detected instance with the highest confidence score was selected as the visualization target, and its predicted category was used as the target class. The gradient of the corresponding class score was backpropagated to the selected feature map. Channel importance weights were obtained by global average pooling of the gradients, after which the weighted feature maps were summed and passed through a ReLU operation. The resulting heatmap was resized to the original image resolution using bilinear interpolation, normalized to the range of [0, 1], and overlaid on the input image. The same target-selection criterion, target layer, and heatmap-generation procedure were used for YOLO11n and DER-YOLO.

**Figure 11:**
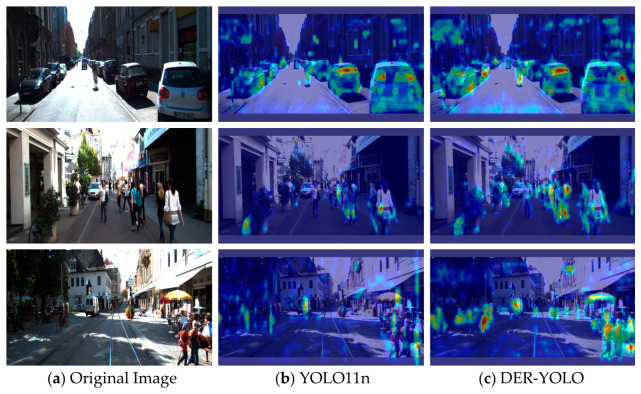
Grad-CAM heatmap comparison on KITTI: (**a**) original image; (**b**) YOLO11n; (**c**) DER-YOLO.

**Figure 12:**
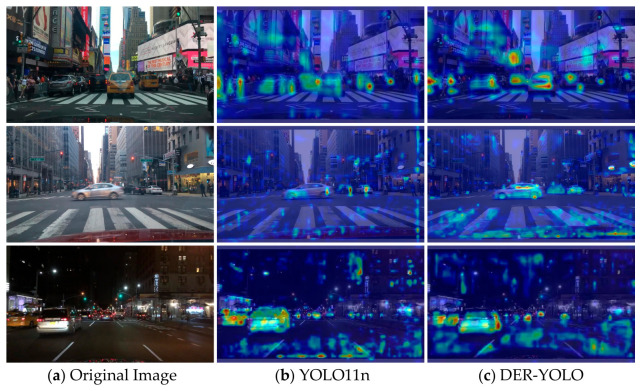
Grad-CAM heatmap comparison on BDD100K: (**a**) original image; (**b**) YOLO11n; (**c**) DER-YOLO.

As shown in Figure 11 and Figure 12, DER-YOLO produces more focused and target-related activation responses than the YOLO11n baseline. In representative KITTI and BDD100K scenes, DER-YOLO shows stronger responses around vehicles, pedestrians, and distant small-object regions, while reducing scattered activations in background areas. Under complex conditions such as dense traffic, low illumination, and nighttime scenes, DER-YOLO also maintains more stable activation responses on traffic-related targets. These visualization results suggest that DER-YOLO can better capture discriminative small-object-related regions and suppress irrelevant background responses, which is consistent with the quantitative scale-wise results and further supports the complementary effects of DGC-C3k2, ECAF, and RLSK.

### 4.8. Error Analysis and Robustness Evaluation

To provide a more objective assessment beyond qualitative visualization, this section reports quantitative error analysis, including robustness under adverse conditions, missed and false detection statistics, and representative failure cases.

#### 4.8.1. Attribute-Subset Evaluation

**Figure 13:**
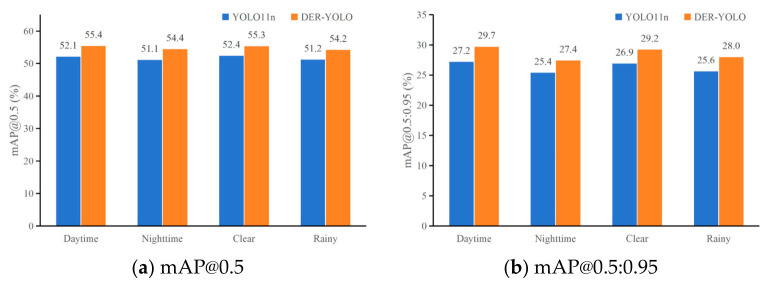
Performance comparison across BDD100K attribute subsets.

To assess robustness under varying illumination and weather conditions, the validation samples were grouped into daytime, nighttime, clear, and rainy subsets. As shown in Figure 13, DER-YOLO consistently achieves higher mAP@0.5 and mAP@0.5:0.95 than YOLO11n across all four subsets. The improvement is stable across conditions, with mAP@0.5 gains ranging from 2.9 to 3.3 percentage points and mAP@0.5:0.95 gains from 2.0 to 2.5 percentage points. Although absolute accuracy is lower under the more challenging nighttime and rainy conditions, DER-YOLO still maintains a clear margin over the baseline in these degraded-visibility settings, indicating that the proposed stage-wise calibration improves robustness without relying on favorable imaging conditions.

#### 4.8.2. Missed and False Detection Analysis

Table 14 compares the FN and FP of YOLO11n and DER-YOLO on the BDD100K validation set. DER-YOLO achieves more true positives while reducing both FP and FN. Specifically, its miss rate decreases from 46.24% to 42.91%, while the recall for small objects increases from 32.40% to 36.85%.

#### 4.8.3. Failure Case Analysis

**Figure 14:**
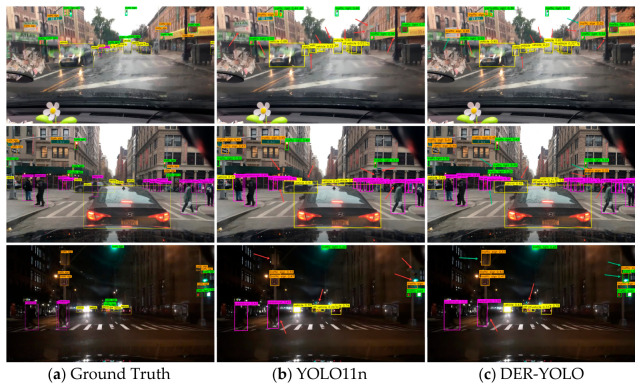
Challenging-case comparison on BDD100K: (**a**) ground truth; (**b**) YOLO11n; (**c**) DER-YOLO. Red arrows denote missed ground-truth targets, whereas green arrows denote targets recovered by DER-YOLO.

To complement the quantitative error analysis, representative challenging cases on the BDD100K validation set are visualized in Figure 14 under three conditions: rainy weather, heavy occlusion, and extreme darkness. For each scene, the ground truth, YOLO11n, and DER-YOLO results are shown side by side. Red arrows indicate ground-truth targets that the corresponding model fails to detect, while green arrows indicate targets that YOLO11n misses but DER-YOLO successfully recovers. As shown in the middle column, YOLO11n leaves many small and low-contrast objects undetected (red arrows), most of which are recovered by DER-YOLO (green arrows in the right column), including distant traffic signs and lights under rain, occluded pedestrians at the intersection, and small dark objects at night. Nevertheless, several targets remain undetected even by DER-YOLO (red arrows in the right column), such as extremely distant vehicles near the vanishing point, heavily overlapped pedestrians in dense crowds, and very dark roadside objects. These observations indicate that, although DER-YOLO substantially improves small-object detection, objects that occupy only a few pixels, are heavily occluded, or appear under very low illumination remain challenging.

## 5. Discussion

### 5.1. Effectiveness of Coordinated Stage-Wise Enhancement

The experimental results indicate that nano-scale YOLO models benefit from coordinated feature calibration across the backbone, neck, and pre-head stages rather than concentrated modification at a single location. DGC-C3k2, ECAF, and RLSK target insufficient context modeling, inadequate cross-scale detail preservation, and limited P3 spatial refinement, respectively. As shown in Table 8, their combination improves mAP@0.5 and mAP@0.5:0.95 by 2.07 and 2.78 percentage points over YOLO11n, exceeding the single-module variants and demonstrating complementary gains. In contrast, the DGC-Net ablation in Table 3 shows that the additional Spatial Gating Branch reduces accuracy, indicating that targeted stage-wise calibration is more effective than indiscriminately increasing module complexity in lightweight onboard detectors.

### 5.2. Small-Object Detection: Progress and Remaining Challenges

The scale-wise analysis in Table 10 further confirms that the improvement brought by DER-YOLO is most evident for small objects, where AP@0.5:0.95 increases by 2.3 percentage points on KITTI and 2.6 percentage points on BDD100K. However, the absolute AP for small objects remains relatively low, indicating that small-object detection in traffic scenes is still challenging. This is mainly because many small targets occupy only a few pixels, making it difficult for the model to extract sufficient discriminative details even after feature enhancement. In addition, when small objects are heavily occluded, spatial calibration alone may be insufficient to recover missing visual cues. These observations suggest that further improvements may require combining stage-wise feature calibration with small-object-oriented data augmentation, higher-resolution training strategies, or temporal information from video sequences.

### 5.3. Limitations and Future Directions

Although DER-YOLO improves detection performance in autonomous driving scenarios, it still has some limitations. First, RLSK is only applied to the P3 branch responsible for small-object detection, whereas the P4 and P5 branches may also require adaptive optimization under challenging conditions such as occlusion, large scale variation, and complex backgrounds. Second, the experiments were conducted on static image datasets, including KITTI and BDD100K, so the temporal consistency of DER-YOLO in video scenes remains unclear. In actual driving, unstable inter-frame detection may affect subsequent tracking and planning; therefore, further evaluation on video benchmark datasets such as nuScenes [51] and Waymo Open [52] is needed. Finally, DER-YOLO has not yet been tested on an in-vehicle embedded platform, and its actual inference speed, power consumption, and deployment stability under hardware constraints still need to be verified. Future work will explore scale-adaptive spatial calibration, multimodal LiDAR–vision fusion [53], video-based evaluation on nuScenes and Waymo Open, and deployment-oriented model optimization for onboard traffic perception systems.

## 6. Conclusions

This paper presents DER-YOLO, a lightweight small-object-oriented traffic detector for complex traffic scenes under onboard vision-based traffic perception conditions. Rather than pursuing general accuracy improvement by increasing model size, DER-YOLO addresses three key bottlenecks that limit small-object detection in nano-scale YOLO models: weak contextual representation, insufficient cross-scale detail preservation, and limited pre-head spatial response calibration. Specifically, DGC-C3k2 enhances global contextual representation after local feature extraction, ECAF adaptively balances high-level semantic cues and shallow high-resolution details in the P3 fusion path, and RLSK refines high-resolution spatial responses before the P3 detection head. These coordinated stage-wise feature calibrations improve small-object representation while preserving the lightweight and real-time characteristics of YOLO11n.

Experiments on KITTI and BDD100K demonstrate that DER-YOLO improves detection accuracy while maintaining real-time inference. On KITTI, small-object AP@0.5:0.95 increases from 27.0% to 29.3%, and on BDD100K it increases from 12.6% to 15.2%, with the largest AP gains consistently observed for small objects. Ablation results further show that distributed and well-positioned enhancements across the detection pipeline are more effective than concentrated modifications at a single stage, while controlling module complexity remains important for lightweight design. Nevertheless, absolute small-object accuracy remains limited, and further optimization is needed for deployment on more constrained edge devices. Future work will explore scale-adaptive spatial calibration, video-based evaluation on nuScenes and Waymo Open datasets, and deployment-oriented model optimization for onboard vision-based traffic perception systems.

## Figures and Tables

**Table 1:** Category-wise instance counts of small, medium, and large objects in the reorganized KITTI and BDD100K datasets. Object scales follow the same COCO criterion as in Figure 5: small, area < 32^2^ pixels; medium, 32^2^ ≤ area < 96^2^ pixels; and large, area ≥ 96^2^ pixels.

Dataset	Class	Small	Medium	Large	Total
KITTI	Car	6288	17,357	9105	32,750
KITTI	Pedestrian	1140	2590	979	4709
KITTI	Cyclist	496	846	285	1627
BDD100K	Vehicle	309,004	275,716	138,943	723,663
BDD100K	Pedestrian	71,556	34,681	3512	109,749
BDD100K	Traffic sign	291,057	63,384	9836	364,277
BDD100K	Traffic light	255,803	25,012	3411	284,226

**Table 2:** Experimental environment and training parameter settings.

Item	Setting
CPU	Intel Core i9-13900H
GPU	NVIDIA GeForce RTX 4060
Framework	PyTorch 2.5.1, CUDA 12.1, Ultralytics 8.3.223
Input size	640 × 640
Batch size	16
Epochs	200
Optimizer	AdamW
Initial learning rate	0.00125
LR schedule	Linear decay (lrf = 0.01)
Momentum	0.9
Weight decay	0.0005
Warm-up epochs	3

**Table 3:** DGC-Net component ablation.

Configuration	α	mAP@0.5 (%)	mAP@0.5:0.95 (%)	Params (M)	GFLOPs
Baseline	-	84.88	58.11	2.590	6.443
C	-	83.82	56.57	2.608	7.028
C+T	-	85.32	58.53	2.621	7.060
C + T + S (Mul)	-	84.82	58.20	2.625	7.084
C + T + S (Res)	0.1	84.52	58.15	2.625	7.084
C + T + S (Res)	0.2	84.61	58.32	2.625	7.084

Note: C denotes Context Modeling, T denotes Transform, and S denotes Spatial Gating. “Mul” and “Res” indicate multiplicative and residual spatial gating, respectively; α is the residual weight coefficient.

**Table 4:** Comparison of DGC-C3k2 with attention and context modules.

Configuration	Position	mAP@0.5 (%)	mAP@0.5:0.95 (%)	Params (M)	GFLOPs
YOLO11n	-	84.88	58.11	2.590	6.443
+SE	Backbone P3 & P4	85.07	58.26	2.595	6.448
+CBAM	Backbone P3 & P4	84.67	57.88	2.595	6.453
+CA	Backbone P3 & P4	84.93	58.15	2.597	6.463
+GCNet	Backbone P3 & P4	85.24	58.37	2.595	6.448
+DGC-C3k2	Backbone P3 & P4	85.32	58.53	2.621	7.060

**Table 5:** Comparison of ECAF upsampling and fusion strategies.

Configuration	mAP@0.5 (%)	mAP@0.5:0.95 (%)	Params (M)	GFLOPs
Fixed (nearest) + concat	84.81	57.97	2.788	8.660
CARAFE + concat	85.12	58.45	2.965	9.224
DySample + concat	85.31	58.71	2.805	8.712
ECAF (DySample + ECA fusion)	85.55	58.38	2.707	7.215

**Table 6:** RLSK branch ablation.

Method	mAP@0.5 (%)	mAP@0.5:0.95 (%)	Params (M)	GFLOPs
Baseline	84.88	58.11	2.590	6.443
3 × 3	85.02	58.35	2.603	6.572
3 × 3/5 × 5	85.15	58.48	2.612	6.644
3 × 3/5 × 5/7 × 7	85.21	58.56	2.644	6.786

**Table 7:** RLSK placement ablation.

Configuration	mAP@0.5 (%)	mAP@0.5:0.95 (%)	Params (M)	GFLOPs
Baseline	84.88	58.11	2.590	6.443
P3	85.21	58.56	2.644	6.786
P4	85.04	58.41	2.679	6.772
P5	84.96	58.29	2.832	6.801
P3 + P4 + P5	85.26	58.68	2.905	7.093

**Table 8:** Ablation results of different module combinations in DER-YOLO.

Method	DGC-C3k2	ECAF	RLSK	*P* (%)	*R* (%)	mAP@0.5 (%)	mAP@0.5:0.95 (%)	FPS	Params (M)	GFLOPs
Baseline ①	-	-	-	89.05	75.01	84.88	58.11	127.2	2.590	6.443
②	✓			89.38	76.26	85.32	58.53	125.9	2.621	7.060
③		✓		88.03	76.78	85.55	58.38	116.8	2.707	7.215
④			✓	89.11	75.35	85.21	58.56	123.4	2.644	6.786
⑤	✓	✓		89.85	76.16	86.33	59.84	114.7	2.724	7.230
DER-YOLO ⑥	✓	✓	✓	91.15	76.66	86.95	60.89	111.4	2.744	7.401

**Table 9:** Effect of pretrained initialization on KITTI.

Method	Init.	mAP@0.5 (%)	mAP@0.5:0.95 (%)
YOLO11n	scratch	84.88	58.11
YOLO11n	pretrained	85.91	59.38
DER-YOLO	scratch	86.95	60.89
DER-YOLO	pretrained	87.72	61.94

**Table 10:** Scale-wise detection performance comparison on KITTI and BDD100K.

Metric	IoU	Area	maxDets	KITTI		BDD100K	
				YOLO11n	DER-YOLO	YOLO11n	DER-YOLO
AP (%)	0.50:0.95	all	100	57.7	60.1	26.1	28.6
	0.50	all	100	83.7	85.9	51.4	53.8
	0.75	all	100	58.0	60.7	22.4	25.0
	0.50:0.95	small	100	27.0	29.3	12.6	15.2
	0.50:0.95	medium	100	54.8	56.2	41.5	43.7
	0.50:0.95	large	100	69.5	70.3	56.9	57.4
AR (%)	0.50:0.95	all	1	25.0	26.2	9.1	9.6
	0.50:0.95	all	10	56.5	59.4	32.5	34.9
	0.50:0.95	all	100	61.2	63.7	40.2	42.0
	0.50:0.95	small	100	40.4	42.7	27.0	28.9
	0.50:0.95	medium	100	63.7	65.3	57.6	59.3
	0.50:0.95	large	100	76.2	76.5	66.7	67.0

**Table 11:** Category-wise small-object AP@0.5:0.95 comparison.

Method	KITTI			BDD100K			
	Car	Pedestrian	Cyclist	Vehicle	Pedestrian	Traffic Sign	Traffic Light
YOLO11n	47.6	12.2	21.2	18.2	11.7	10.6	9.9
DER-YOLO	48.9	14.4	24.6	19.9	14.0	13.9	13.0

**Table 12:** Performance comparison on KITTI.

Method	mAP@0.5 (%)	mAP@0.5:0.95 (%)	FPS	Params (M)	GFLOPs
NanoDet-Plus-m	84.22	55.17	115.4	1.168	3.695
YOLOv8n	85.02	58.22	118.9	3.012	8.197
YOLOv10n	82.98	57.31	109.9	2.709	8.399
YOLO11n	84.88	58.11	127.2	2.590	6.443
YOLO11s	88.20	63.06	94.6	9.429	21.600
YOLOv12n	85.32	58.34	121.1	2.569	6.483
HIC-YOLOv5	91.46	62.89	76.4	9.314	31.000
RT-DETR-r18	86.40	59.11	59.2	19.921	57.085
DER-YOLO	86.95	60.89	111.4	2.744	7.401

Note: YOLO11n and DER-YOLO were each trained three times with random seeds 42, 43, and 44 under identical settings. The table reports a representative single run for consistency with the ablation studies. Across the three runs, the standard deviations are ±0.14/±0.10 (mAP@0.5/mAP@0.5:0.95) for YOLO11n and ±0.16/±0.12 for DER-YOLO, and the representative single run reported above lies within one standard deviation of the corresponding three-run mean. Other methods are from a single run.

**Table 13:** Performance comparison on BDD100K.

Method	mAP@0.5 (%)	mAP@0.5:0.95 (%)	FPS	Params (M)	GFLOPs
NanoDet-Plus-m	51.38	25.12	110.6	1.168	3.695
YOLOv8n	52.00	26.42	115.4	3.012	8.197
YOLOv10n	51.43	26.22	105.2	2.709	8.399
YOLO11n	52.05	26.51	122.8	2.590	6.443
YOLO11s	55.68	29.56	89.5	9.429	21.600
YOLOv12n	52.61	26.75	118.1	2.569	6.483
HIC-YOLOv5	60.62	29.30	71.2	9.314	31.000
RT-DETR-r18	54.48	28.52	56.2	19.921	57.085
DER-YOLO	55.05	29.21	106.3	2.744	7.401

Note: YOLO11n and DER-YOLO were each trained three times with random seeds 42, 43, and 44 under identical settings. The table reports a representative single run for consistency with the ablation studies. Across the three runs, the standard deviations are ±0.19/±0.11 (mAP@0.5/mAP@0.5:0.95) for YOLO11n and ±0.21/±0.13 for DER-YOLO, and the representative single run reported above lies within one standard deviation of the corresponding three-run mean. Other methods are from a single run.

**Table 14:** Quantitative error and scale-wise recall analysis on BDD100K.

Model	TP	FP	FN	Miss Rate (%)	R-Small (%)
YOLO11n	99,748	26,383	85,778	46.24	32.40
DER-YOLO	105,915	24,799	79,611	42.91	36.85

Note: Miss rate is calculated as FN/(TP + FN), and R-small denotes recall on small objects. The TP, FP, and FN counts are obtained at a confidence threshold of 0.25 and an IoU matching threshold of 0.5.

## Data Availability

The data used in this study were obtained from publicly available benchmark datasets. The KITTI dataset is available from the KITTI Vision Benchmark Suite at https://www.cvlibs.net/datasets/kitti/ (accessed on 15 February 2026). The BDD100K dataset is available from the Berkeley DeepDrive platform at https://deepdrive.berkeley.edu/ (accessed on 15 February 2026), and related dataset information and tools are also provided at https://github.com/bdd100k/bdd100k (accessed on 15 February 2026). The datasets are available for academic research purposes under their respective terms of use. The core implementation code of DER-YOLO, including the DGC-C3k2, ECAF, and RLSK modules, together with the model configuration files for KITTI and BDD100K, is publicly available at https://github.com/weijp528/DER-YOLO (accessed on 2 June 2026).

## References

[1] Song Z., Liu L., Jia F., Luo Y., Zhang G., Yang L., Wang L., Jia C. (2024). Robustness-Aware 3D Object Detection in Autonomous Driving: A Review and Outlook. IEEE Trans. Intell. Transp. Syst..

[2] Liu Z., Wu J., Cai Y., Wang H., Chen L., Liu Q. (2025). Dual-Stage Feature Specialization Network for Robust Visual Object Detection in Autonomous Vehicles. Sci. Rep..

[3] Yue S., Zhang Z., Shi Y., Cai Y. (2024). WGS-YOLO: A Real-Time Object Detector Based on YOLO Framework for Autonomous Driving. Comput. Vis. Image Underst..

[4] Wei J., As’arry A., Anas Md Rezali K., Zuhri Mohamed Yusoff M., Ma H., Zhang K. (2025). A Review of YOLO Algorithm and Its Applications in Autonomous Driving Object Detection. IEEE Access.

[5] Ruan J., Cui H., Huang Y., Li T., Wu C., Zhang K. (2023). A Review of Occluded Objects Detection in Real Complex Scenarios for Autonomous Driving. Green Energy Intell. Transp..

[6] Redmon J., Divvala S., Girshick R., Farhadi A. (2016). You Only Look Once: Unified, Real-Time Object Detection. Proceedings of the 2016 IEEE Conference on Computer Vision and Pattern Recognition (CVPR), Las Vegas, NV, USA, 27–30 June 2016.

[7] Khanam R., Hussain M. (2024). YOLOv11: An Overview of the Key Architectural Enhancements. arXiv.

[8] Jocher G., Qiu J. Ultralytics YOLO11. https://docs.ultralytics.com/models/yolo11/.

[9] Tian Y., Ye Q., Doermann D. (2025). YOLOv12: Attention-Centric Real-Time Object Detectors. arXiv.

[10] Zheng Z., Cheng J., Jin F. (2025). TFP-YOLO: Obstacle and Traffic Sign Detection for Assisting Visually Impaired Pedestrians. Sensors.

[11] Liang E., Wei D., Li F., Lv H., Li S. (2025). Object Detection Model of Vehicle-Road Cooperative Autonomous Driving Based on Improved YOLO11 Algorithm. Sci. Rep..

[12] Redmon J., Farhadi A. (2018). YOLOv3: An Incremental Improvement. arXiv.

[13] Jocher G., Stoken A., Borovec J. Ultralytics YOLOv5. https://zenodo.org/records/7347926.

[14] Wang C.-Y., Mark Liao H.-Y., Wu Y.-H., Chen P.-Y., Hsieh J.-W., Yeh I.-H. (2020). CSPNet: A New Backbone That Can Enhance Learning Capability of CNN. Proceedings of the 2020 IEEE/CVF Conference on Computer Vision and Pattern Recognition Workshops (CVPRW), Seattle, WA, USA, 14–19 June 2020.

[15] Liu S., Qi L., Qin H., Shi J., Jia J. (2018). Path Aggregation Network for Instance Segmentation. Proceedings of the 2018 IEEE/CVF Conference on Computer Vision and Pattern Recognition (CVPR), Salt Lake City, UT, USA, 18–22 June 2018.

[16] Wang C.-Y., Bochkovskiy A., Liao H.-Y.M. (2023). YOLOv7: Trainable Bag-of-Freebies Sets New State-of-the-Art for Real-Time Object Detectors. Proceedings of the 2023 IEEE/CVF Conference on Computer Vision and Pattern Recognition (CVPR), Vancouver, BC, Canada, 17–24 June 2023.

[17] Jocher G., Chaurasia A., Qiu J. Ultralytics YOLOv8. https://zenodo.org/records/13614592.

[18] Wang A., Chen H., Liu L., Chen K., Lin Z., Han J., Ding G. (2024). YOLOv10: Real-Time End-to-End Object Detection. Adv. Neural Inf. Process. Syst..

[19] Zhao Y., Lv W., Xu S., Wei J., Wang G., Dang Q., Liu Y., Chen J. (2024). DETRs Beat YOLOs on Real-Time Object Detection. Proceedings of the 2024 IEEE/CVF Conference on Computer Vision and Pattern Recognition (CVPR), Seattle, WA, USA, 16 June 2024.

[20] Zhang W., Gao X., Yang C., Jiang F., Chen Z. (2022). A Object Detection and Tracking Method for Security in Intelligence of Unmanned Surface Vehicles. J. Ambient Intell. Hum. Comput..

[21] Chen G., Wang H., Chen K., Li Z., Song Z., Liu Y., Chen W., Knoll A. (2022). A Survey of the Four Pillars for Small Object Detection: Multiscale Representation, Contextual Information, Super-Resolution, and Region Proposal. IEEE Trans. Syst. Man Cybern. Syst..

[22] Cheng G., Yuan X., Yao X., Yan K., Zeng Q., Xie X., Han J. (2023). Towards Large-Scale Small Object Detection: Survey and Benchmarks. IEEE Trans. Pattern Anal. Mach. Intell..

[23] Li T., Xiong X., Zhang Y., Fan X., Zhang Y., Huang H., Hu D., He M., Liu Z. (2025). RE-YOLOv5: Enhancing Occluded Road Object Detection via Visual Receptive Field Improvements. Sensors.

[24] Yang Y., Yang S., Chan Q. (2025). LEAD-YOLO: A Lightweight and Accurate Network for Small Object Detection in Autonomous Driving. Sensors.

[25] He X., Cheng R., Zheng Z., Wang Z. (2021). Small Object Detection in Traffic Scenes Based on YOLO-MXANet. Sensors.

[26] Khalili B., Smyth A.W. (2024). SOD-YOLOv8—Enhancing YOLOv8 for Small Object Detection in Aerial Imagery and Traffic Scenes. Sensors.

[27] Ding X., Zhang X., Han J., Ding G. (2022). Scaling Up Your Kernels to 31x31: Revisiting Large Kernel Design in CNNs. Proceedings of the IEEE/CVF Conference on Computer Vision and Pattern Recognition (CVPR), New Orleans, LO, USA, 19–24 June 2022.

[28] Guo M.-H., Lu C.-Z., Liu Z.-N., Cheng M.-M., Hu S.-M. (2023). Visual Attention Network. Comput. Vis. Media.

[29] Li X., Wang W., Hu X., Yang J. (2019). Selective Kernel Networks. Proceedings of the 2019 IEEE/CVF Conference on Computer Vision and Pattern Recognition (CVPR), Long Beach, CA, USA, 15–20 June 2019.

[30] Li Y., Hou Q., Zheng Z., Cheng M.-M., Yang J., Li X. (2023). Large Selective Kernel Network for Remote Sensing Object Detection. Proceedings of the 2023 IEEE/CVF International Conference on Computer Vision (ICCV), Paris, France, 1 October 2023.

[31] Lau K.W., Po L.-M., Rehman Y.A.U. (2024). Large Separable Kernel Attention: Rethinking the Large Kernel Attention Design in CNN. Expert Syst. Appl..

[32] Hu J., Shen L., Albanie S., Sun G., Wu E. Squeeze-and-Excitation Networks. Proceedings of the IEEE/CVF Conference on Computer Vision and Pattern Recognition (CVPR).

[33] Woo S., Park J., Lee J.-Y., Kweon I.S., Ferrari V., Hebert M., Sminchisescu C., Weiss Y. (2018). CBAM: Convolutional Block Attention Module. Proceedings of the Computer Vision—ECCV 2018.

[34] Wang X., Girshick R., Gupta A., He K. (2018). Non-Local Neural Networks. Proceedings of the 2018 IEEE/CVF Conference on Computer Vision and Pattern Recognition, Salt Lake City, UT, USA, 18–22 June 2018.

[35] Cao Y., Xu J., Lin S., Wei F., Hu H. (2019). GCNet: Non-Local Networks Meet Squeeze-Excitation Networks and Beyond. Proceedings of the 2019 IEEE/CVF International Conference on Computer Vision Workshop (ICCVW), Seoul, Republic of Korea, 27–28 October 2019.

[36] Hou Q., Zhou D., Feng J. (2021). Coordinate Attention for Efficient Mobile Network Design. Proceedings of the 2021 IEEE/CVF Conference on Computer Vision and Pattern Recognition (CVPR), Nashville, TN, USA, 20–25 June 2021.

[37] Shi H., Wu Y., Xu Y., Mu X., Hou M., Shi B., Zhang L. (2026). MDI-YOLO a Lightweight Transformer-CNN-Based Multidimensional Feature Fusion Model for Small Object Detection. Sci. Rep..

[38] Yang J., Yue X., Wu L. (2026). A Collaborative Multi-Attention Network for Real-Time Small Object Detection in UAV Imagery. Sci. Rep..

[39] Lin T.-Y., Dollár P., Girshick R., He K., Hariharan B., Belongie S. (2017). Feature Pyramid Networks for Object Detection. Proceedings of the 2017 IEEE Conference on Computer Vision and Pattern Recognition (CVPR), Honolulu, HI, USA, 21–26 July 2017.

[40] Tan M., Pang R., Le Q.V. (2020). EfficientDet: Scalable and Efficient Object Detection. Proceedings of the 2020 IEEE/CVF Conference on Computer Vision and Pattern Recognition (CVPR), Seattle, WA, USA, 13–19 June 2020.

[41] Ghiasi G., Lin T.-Y., Le Q.V. (2019). NAS-FPN: Learning Scalable Feature Pyramid Architecture for Object Detection. Proceedings of the 2019 IEEE/CVF Conference on Computer Vision and Pattern Recognition (CVPR), Long Beach, CA, USA, 15–20 June 2019.

[42] Wang J., Chen K., Xu R., Liu Z., Loy C.C., Lin D. (2019). CARAFE: Content-Aware ReAssembly of FEatures. Proceedings of the 2019 IEEE/CVF International Conference on Computer Vision (ICCV), Seoul, Republic of Korea, 27 October–2 November 2019.

[43] Liu W., Lu H., Fu H., Cao Z. Learning to Upsample by Learning to Sample. Proceedings of the IEEE/CVF International Conference on Computer Vision (ICCV).

[44] Wang Q., Wu B., Zhu P., Li P., Zuo W., Hu Q. (2020). ECA-Net: Efficient Channel Attention for Deep Convolutional Neural Networks. Proceedings of the 2020 IEEE/CVF Conference on Computer Vision and Pattern Recognition (CVPR), Seattle, WA, USA, 14–19 June 2020.

[45] Geiger A., Lenz P., Urtasun R. (2012). Are We Ready for Autonomous Driving? The KITTI Vision Benchmark Suite. Proceedings of the 2012 IEEE Conference on Computer Vision and Pattern Recognition, Providence, RI, USA, 16–21 June 2012.

[46] Yu F., Chen H., Wang X., Xian W., Chen Y., Liu F., Madhavan V., Darrell T. (2020). BDD100K: A Diverse Driving Dataset for Heterogeneous Multitask Learning. Proceedings of the 2020 IEEE/CVF Conference on Computer Vision and Pattern Recognition (CVPR), Seattle, WA, USA, 13–19 June 2020.

[47] Lin T.-Y., Maire M., Belongie S., Hays J., Perona P., Ramanan D., Dollár P., Zitnick C.L., Fleet D., Pajdla T., Schiele B., Tuytelaars T. (2014). Microsoft COCO: Common Objects in Context. Computer Vision—ECCV 2014.

[48] Rangi L. NanoDet-Plus: Super Fast and High-Accuracy Lightweight Anchor-Free Object Detection Model. https://github.com/RangiLyu/nanodet.

[49] Tang S., Zhang S., Fang Y. HIC-YOLOv5: Improved YOLOv5 for Small Object Detection. Proceedings of the 2024 IEEE International Conference on Robotics and Automation (ICRA).

[50] Selvaraju R.R., Cogswell M., Das A., Vedantam R., Parikh D., Batra D. Grad-CAM: Visual Explanations From Deep Networks via Gradient-Based Localization. Proceedings of the 2017 IEEE International Conference on Computer Vision (ICCV).

[51] Caesar H., Bankiti V., Lang A.H., Vora S., Liong V.E., Xu Q., Krishnan A., Pan Y., Baldan G., Beijbom O. (2020). nuScenes: A Multimodal Dataset for Autonomous Driving. Proceedings of the 2020 IEEE/CVF Conference on Computer Vision and Pattern Recognition (CVPR), Seattle, WA, USA, 14–19 June 2020.

[52] Sun P., Kretzschmar H., Dotiwalla X., Chouard A., Patnaik V., Tsui P., Guo J., Zhou Y., Chai Y., Caine B. (2020). Scalability in Perception for Autonomous Driving: Waymo Open Dataset. Proceedings of the 2020 IEEE/CVF Conference on Computer Vision and Pattern Recognition (CVPR), Seattle, WA, USA, 13–19 June 2020.

[53] Zhang W., Jiang F., Yang C.-F., Wang Z.-P., Zhao T.-J. (2021). Research on Unmanned Surface Vehicles Environment Perception Based on the Fusion of Vision and Lidar. IEEE Access.

